# Prevalence, trends and associated factors of malaria in the Shai-Osudoku District Hospital, Ghana

**DOI:** 10.1186/s12936-023-04561-y

**Published:** 2023-04-22

**Authors:** Jessica Ashiakie Tetteh, Patrick Elorm Djissem, Alfred Kwesi Manyeh

**Affiliations:** 1 Fred N Binka School of Public Health, University of Health and Allied Sciences, Hohoe, Volta Region Ghana; 2 Institute of Health Research, University of Health and Allied Sciences, Ho, Volta Region Ghana

**Keywords:** Malaria, Prevalence, Trend, Shai-Osudoku District Hospital, Ghana

## Abstract

**Background:**

Even though malaria is easily preventable and treatable, it continues to have a devastating impact on people’s health and livelihoods around the world. Sub-Saharan Africa carries a disproportionately high share of the global malaria burden. This study seeks to assess the prevalence, trends and factors associated with malaria in the Shai-Osudoku District Hospital, Ghana.

**Methods:**

A cross-sectional study was conducted to determine the prevalence, trend, and factors associated with malaria in the Shai-Osudoku District Hospital; a 10-month secondary data was extracted from February to November 2020. The extracted data were entered into Epi Data version 6 and analysed using STATA version 16. Descriptive analysis was performed to determine the prevalence, trend and socio-demographic characteristics of study participants. Simple logistic regression at a 95% confidence level was performed to investigate socio-demographic factors associated with malaria infection. Tables and charts with summary statistics were used to present the results.

**Results:**

Secondary data from 3896 individuals were included in the study. The age of the participants range from 0.8 to 101 years with a mean age of 32.5. The estimated prevalence of malaria during the study period is 20.9%. A majority (79.1%) of the participants who presented signs and symptoms of malaria were negative after testing. The prevalence of malaria cases increased progressively from 6.7 to 55.4% across the ten months. The simple logistic regression at a 95% confidence level revealed that age group, sex, residential status, religion, occupation and marital status were statistically significantly associated with malaria. The results shows that persons who tested positive for malaria were mostly treated with artemether-lumefantrine (46.1%), some malaria positive cases were given artesunate injection (11.6%), dihydroartemisinin-piperaquine (16.2%) and oral artemether-lumefantrine (6.5%). Surprisingly 19.6% of the malaria-positive cases were not given any form of malaria medication.

**Conclusion:**

Factors found to influence malaria infection in the Shai-Osudoku District Hospital include participant’s age, sex, residential status, religious affiliation occupation and marital status. The findings of this study showed that malaria remains a serious public health problem in the Shai Osudoku District Hospital. The information obtained from this study can guide the implementation of malaria prevention, control and elimination strategies in Ghana.

## Background

Malaria continues to be endemic and perennial in all parts of Ghana, with seasonal variations that are more pronounced in the north despite the efforts by the governments of Ghana to mitigate its prevalence [1]. Children under five years of age and pregnant women are at higher risk of severe illness due to lowered immunity [2]. Malaria remains one of the leading causes of morbidity, accounting for about 38 per cent of all outpatient illnesses, 35 per cent of all admissions, and about 34 percent of all deaths in children under five years of age [3].

Long-lasting insecticidal nets (LLINs) are one of the most efficacious preventive interventions against malaria morbidity and mortality available [4] and form a cornerstone of the Roll Back Malaria (RBM) Partnership’s scaling-up for impact strategy to reduce malaria-related mortality by 75 percent by 2015 [5]. This study seeks to assess the prevalence, trend and factors associated with malaria in the Shai-Osudoku District Hospital of Ghana.

## Methods

### Study design

A cross-sectional study was conducted. Secondary data were extracted from hospital records covering ten months (February 2020 to November 2020) in the Shai-Osudoku District Hospital (SODH), Ghana. All records of patients who visited SODH within the study period and were requested to go for a malaria test were extracted.

### Study site description

The study was conducted in the Shai-Osudoku District Hospital (SODH). The SODH was established in 1970 as a health post by Shai and handed over to the Ministry of Health. The facility advanced to a Health Centre in 1985 and finally to a district hospital in mid-2009. The hospital is situated in Dodowa, the capital of the Shai-Osudoku District. The hospital is the only major government health institution in the Shai-Osudoku District. It is a 125-bed capacity hospital with six wards, two operating theatres, a laboratory and a physiotherapy unit. The Shai-Osudoku District is one of the 260 Metropolitan, Municipal and Districts Assemblies in Ghana and forms part of the 29 Metropolitan, Municipal and District Assemblies (MMDAs) in the Greater Accra Region. The SODH is in the southeastern part of Ghana, lying between latitude 5.45º South and 6.05º North and Longitude 0.05º East and 0.20º West. The district was carved out of the former Dangme West District [6]. In all, the district occupies a total land area of about 968.361 square kilometers. Based on Legislative Instrument (LI) 2137, Dangme West District was split into two in June 2012 to have Ningo Prampram District and Shai-Osudoku District. It shares boundaries with the North Tongu District to the North-East, Yilo and Lower Manya Districts to the North-West, Akwapim North District to the West, Kpone-Kantamanso District to the South West, Ningo Prampram District to the South and the Ada West District to the East. The Volta River washes the North-Eastern portions of the district. The district has a hospital, three health centres, eight Community-based Health Planning Services (CHPS) compounds, seventeen CHPS zones, and four private facilities [6].

The Shai Osudoku district was specifically selected because it is among the Greater Accra region districts where malaria is endemic. The National Malaria Control Programme also carried out a free bed net distribution in all district communities in 2018.

### Sample size determination

All persons who visited the hospital during February and November 2020 and were requested to take a malaria test were included in the study. The secondary socio-demographic and laboratory data of these patients were extracted from the hospital records.

### Sampling method

This is a secondary data extraction, and there was no sampling in this study. The hospital records of all patients who met the inclusion criteria were extracted and included in the study for analysis. The data of 3896 participants were extracted and included in the study.

### Inclusion and exclusion criteria

Data of all patients who visited SODH and conducted a malaria test between February 2020 to November 2020 irrespective of sex and age was extracted and included in the study. Data of patients who visited SODH before February 2020 and after November 2020 was excluded from the study. All patients who visited SODH within the study period but did not conduct malaria test was also excluded from the study.

### Data collection procedure

At the district hospital, data on malaria covering the study period in the laboratory’s registers (date the test was done, patients age, and test results) and in the patients' folders (patients’ sex, occupation, marital status, address, and type of treatment given) was extracted. Subsequently, data were extracted and recorded chronologically with the help of a pre-tested questionnaire. Each extracted data from the laboratory register was linked to the respective folder using the patient folder number. Data included the year test was done, the month, the patient's age, sex, occupation, type of treatment, location, marital status, and laboratory results in trophozoite/µl of blood. The data was extracted and recorded chronologically with the help of a pre-tested questionnaire.

### Variables and statistical analysis

The outcome variable is malaria status and it was coded as 1 for a positive malaria test and 0 if otherwise. The explanatory variables include age, sex, residential status, religion, occupation, and marital status. These were coded as categorical variables. The extracted data were entered into Epi Data version 6. The data is imported into STATA 16 for cleaning and analysis. Descriptive analysis was performed to determine the prevalence, trend and socio-demographic characteristics of study participants. Simple logistic regression at a 95% confidence level was performed to investigate socio-demographic factors associated with malaria infection among the study participants. The results were presented in tables and charts with summary statistics.

### Ethical considerations

Ethical approval was obtained from the University of Health and Allied Sciences Ethical Review Committee before the commencement of the study; with approval number: UHAS-REC A.9 [82] 20–21. Permission was sought from the Shai-Osudoku District Health Directorate and the management of the Shai-Osudoku District Hospital. Confidentiality was observed throughout the study.

## Results

### Socio-demographic characteristics

Table 1 presents the Socio-demographic characteristics of 3896 study participants. The ages of the participants ranged from 0.08 to 101 years with a mean of 32.5. Females were in majority (70.7%) and males constituted only 29.3% of the study participants. A large proportion of the study participants 69.3% resided in peri-urban areas. Christians accounted for 74.3% while Muslims constituted 18.1% and 7.6% of the participants had no religious affiliation. While 23.5% of the study participants were unemployed, 69.8% were employed.

**Table 1:** Socio-demographic characteristics of participants

Variables	Frequency [n = 3896]	Percent (%)
Age in complete years		
≤ 10	402	10.3
11–20	393	10.1
21–30	1096	28.1
31–40	968	24.9
41–50	507	13.0
≥ 51	530	13.6
Mean age (32.5 ± 17.4)		
Sex		
Female	2754	70.7
Male	1142	29.3
Residential status		
Urban	2700	69.3
Rural	1196	30.7
Religion		
No Religion	295	7.6
Christian	2895	74.3
Muslim	706	18.1
Occupation		
Unemployed	916	23.5
Employed	2720	69.8
Missing	260	6.7
Marital status		
Single	1834	47.1
Married	1771	45.5
Widowed/Divorced/ Separated	104	2.7
Missing	187	4.8

### Prevalence of malaria

**Fig. 1:**
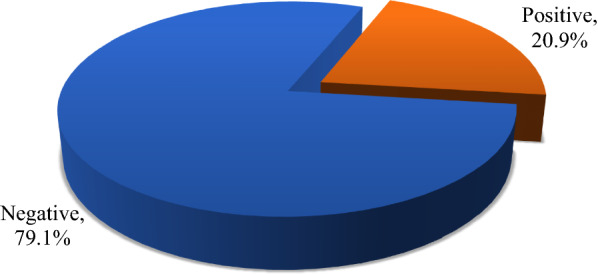
Prevalence of malaria in Shai-Osudoku District Hospital in 2020

The prevalence of malaria in our study was 20.9%. Nonetheless, the majority (79.1%) of the sample whopresented signs and symptoms of malaria were negative after testing (Fig. 1).

### The trend of malaria cases in Shai-Osudoku District Hospital

**Fig. 2:**
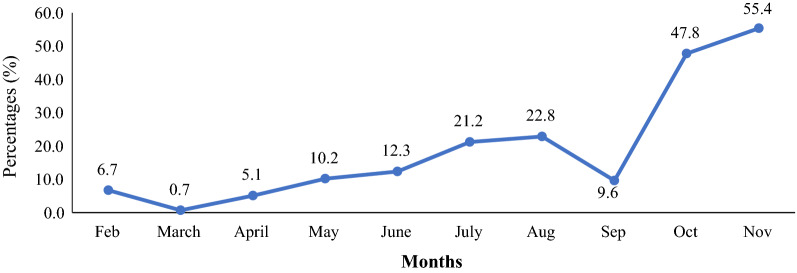
Monthly trend of malaria prevalence in Shai-Osudoku District

Figure 2 reveals that malaria cases increased progressively from 6.7% to 55.4% from February to November 2020. However, malaria cases in March and September reduced to 0.7% and 9.6% respectively. Yet there was a significant rise (47.8% and 55.4%) in October and November, respectively.

### Crude and adjusted odds ratios of socio-demographic determinants of malaria status

Table 2 presents the Crude Odds Ratio (COR) and Adjusted Odds Ratios (AOR) at a 95% Confidence Interval (CI) of sociodemographic characteristics and malaria status. In the crude model, age was found to have a statistically significant association with positive malaria status. Study participants aged 11–20 years were 84% less likely to test positive for malaria (COR: 0.84, 95% CI: 0.62–1.14) as compared to participants aged 10 years and below, while those aged 51 years and above were 46% less likely to test positive for malaria (COR: 0.46, 95% CI: 0.34–0.63). Participants of age groups 21–30, 31–40 and 41–50 were 54%, 47% and 53% less likely to test positive for malaria as compared to participants aged 10 years and below, respectively (COR: 0.54, 95% CI: 0.42–0.70), (COR:0.47, 95% CI: 0.36–0.61), (COR:0.53, 95% CI: 0.39–0.72). Male participants were 44% more likely to test positive for malaria as compared to the female participants (COR: 1.44, 95% CI: 1.22–1.70) and this is statistically significant.

**Table 2:** Crude and adjusted odds ratios of socio-demographic characteristics (factors) and malaria (Microscopy)

Variables	COR (95% CI)	P-value	AOR (95% CI)	P-value
Age in years				
≤ 10	Ref.		Ref.	
11–20	0.84(0.62–1.14)	0.265	0.73(0.52–1.02)	0.068
21–30	0.54(0.42–0.70)	< 0.001*	0.45(0.31–0.65)	< 0.001*
31–40	0.47(0.36–0.61)	< 0.001*	0.42(0.28–0.63)	< 0.001*
41–50	0.53(0.39–0.72)	< 0.001*	0.48(0.31–0.75)	0.001*
≥ 51	0.46(0.34–0.63)	< 0.001*	0.45(0.29–0.71)	0.001*
Sex				
Female	Ref.		Ref.	
Male	1.44(1.22–1.70)	< 0.001*	1.36(1.15–1.61)	< 0.001*
Residential status				
Urban	Ref.		Ref.	
Rural	1.31(1.12–1.55)	0.001*	1.30(1.10–1.53)	0.002*
Religion				
No Religion	Ref.		Ref.	
Christian	0.57(0.44–0.74)	< 0.001*	0.60(0.45–0.78)	< 0.001*
Muslim	0.75(0.55–1.01)	0.060	0.76(0.56–1.04)	0.082
Occupation				
Unemployed	Ref.		Ref.	
Employed	0.84(0.70–1.00)	0.051	1.60(1.21–2.11)	0.001*
Marital status				
Single	Ref.		Ref.	
Married	0.66(0.57–0.78)	< 0.001*	0.78(0.64–0.97)	0.023*
Widowed/Divorced/ Separated	0.58(0.34–0.99)	0.045*	0.73(0.40–1.30)	0.285

*AOR* Adjusted Odds Ratio, *COR* Crude Odds Ratio, *Statistical significance

The crude model further revealed that both residential status and religious affiliation are associated with participants’ malaria status. The odds of testing positive for malaria among rural residents were 31% more as compared to urban residents (COR: 1.31, 95% CI: 1.12–1.55) and this is statistically significant. Also, Christians and Muslims were 57% and 75% less likely to test positive for malaria as compared to those with no religious affiliation respectively (COR: 0.57, 95% CI: 0.44–0.74), (COR: 0.75, 95% CI: 0.55–1.01). Participants who were married were 66% less likely to test positive for malaria compared to those who are single (COR: 0.66, 95% CI: 0.57–0.78) and this is statistically significant. Participants who were separated/divorced/widowed were 58% less likely to test positive for malaria (COR: 0.58, 95% CI: 0.34–0.99) compare to those who were single and this is also statistically significant.

After adjusting for other explanatory variables, participants’ age was found to be statistically significantly associated with testing positive for malaria. Participants aged 21–30, 31–40, 41–50 and 51 + were 45, 42, 48, and 45% less likely to test positive for malaria infection, respectively (AOR: 0.45, 95% CI: 0.31–0.65), (AOR: 0.42, 95% CI: 0.28–0.63), (AOR: 0.48, 95% CI: 0.31–0.75), (AOR: 0.45, 95% CI: 0.29–0.71) and these are statistically significant. Both sex and residential status had statistically significant associations with malaria status; with an increased odds of 36% and 30% of testing positive for malaria compared to females (AOR: 1.36, 95% CI: 1.15–1.61), (AOR: 1.30, 95% CI: 1.10–1.53) and urban residents respectively. The adjusted model revealed that while the odds of testing positive for malaria among Christian participants reduced by 60% compared to those not affiliated with any religion (AOR: 0.60, 95% CI: 0.45–0.78) and this is statistically significant. Muslim participants were 77% less likely to test positive for malaria but this was not statistically significant (AOR: 0.76, 95% CI: 0.56–1.04). The odds of testing positive for malaria also increased by 60% among the participants that were employed (AOR: 1.60, 95% CI: 1.21–2.11) and this is also statistically significant. Again, the odds of being positive for malaria among the participants who married and those either Widowed/Divorced/Separated were 78% (AOR: 0.78, 95% CI: 0.64–0.97) and 73% (AOR: 0.73, 95% CI: 0.40–1.30) less likely, compared to single participants.

### Type of malaria treatments

**Fig. 3:**
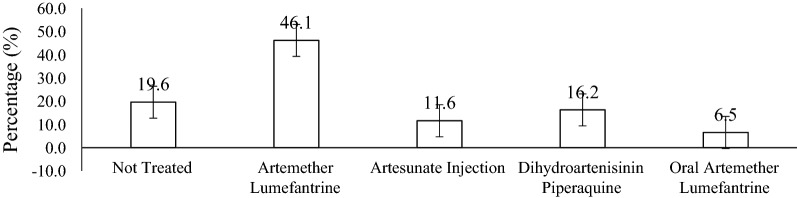
Malaria treatment given to the positive cases

**Fig. 4:**
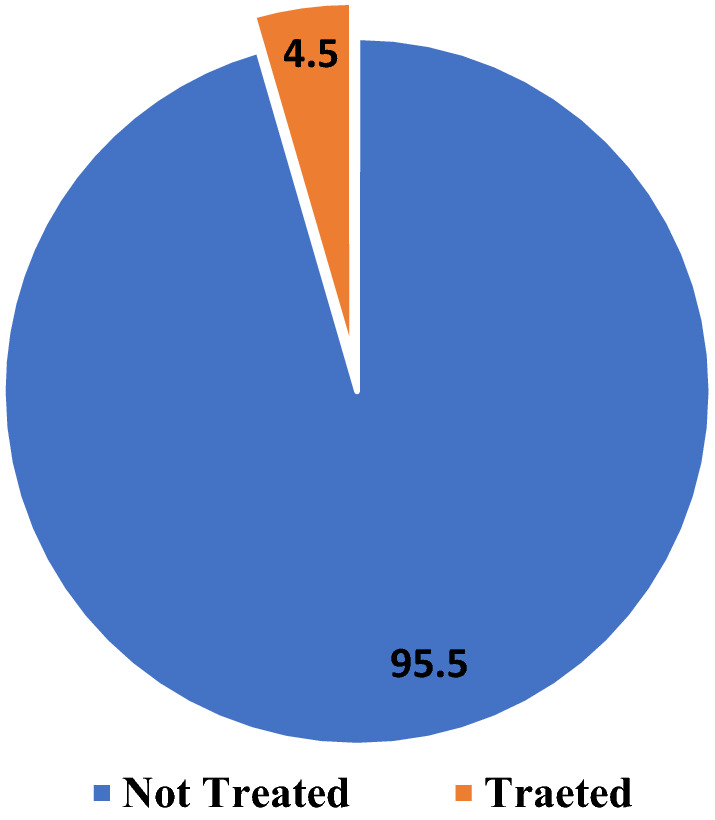
Treatments given to negative cases

Figure 3 shows the type of malaria treatments or drugs given to malaria-positive patients at the Shai-Osudoku District Hospital. Artemether lumefantrine was mostly given (46.1%). Some malaria-positive cases were also treated with Artesunate injections (11.6%). Dihydroartenisinin piperaquine (16.2%) and oral artemether-lumefantrine (6.5%). Surprisingly, (19.6%) of the malaria-positive cases were not given any form of malaria medication. Figure 4 shows that 95.5% of negative participants did not receive any malaria treatment while 4.5% of the participants received malaria treatment even though they tested negative.

## Discussion

Despite the interventions and financial investments in the effort to control and eliminate malaria, the disease continues to be the most serious public health problem and threat to people’s health in sub-Saharan Africa, including Ghana. This study assessed the prevalence, trends, prescribed treatments, and socio-demographic determinants of malaria infection at Shai-Osudoku District Hospital of Ghana.

### Prevalence of malaria cases

Malaria remains a global pressing issue despite efforts to reduce its morbidity and mortality. According to the Ghana demographic and health survey, malaria prevalence ranges from 11.2 to 40.0% [7]. In this study, the prevalence of malaria was found to be 20.9%. This is consistent with a study conducted in Ghana on the prevalence of malaria-positive rapid diagnostic tests and antimalarial treatment which reported a 21.58% prevalence rate [8]. A similar study by Deku and colleagues in their retrospective study on malaria in the Western North region of Ghana reported, a slightly higher prevalence rate of 26.5% was reported [9]. A study conducted on knowledge and prevalence of Malaria among rural households in Ghana reported a prevalence rate of 39.1% [10]. A higher prevalence rate of 58.0% was reported in the Brong-Ahafo region of Ghana [11]. The high prevalence documented in the country is also consistent with studies in other African countries. A study in Nigeria recorded a prevalence of 72.8% [12] and another study in Kenya reported a prevalence rate of 28% [13].

### The trend of malaria cases

Although Ghana is considered to be in the control phase, the malaria incident rate continues to rise annually in many parts of the country [1]. The trend of malaria cases in this study increased progressively from 6.7 to 55.4% from February to November with significant rises in October (47.8%) and November (55.4%). This is a clear indication of the malaria seasonal pattern in the Greater Accra Region with a higher number of cases recorded in the rainy seasons according to Donovan and colleagues [14]. This study revealed that the overall malaria prevalence was 20.9. This could be attributed to the relative increase in the number of breeding grounds for the *Anopheles* mosquitoes, poor control measures, population densities, overcrowding and unhygienic conditions according as established elsewhere [15].

### Factors associated with malaria

Participants’ sex, age, residential status, religion, occupation and marital status were found to be statistically significantly associated with malaria infection in this study. In this current study, male patients were more likely to test positive for malaria as compared to females. This finding is consistent with the result of a study from Cameroon which established that the prevalence of malaria was observed to be significantly associated with gender with males having a higher prevalence compared to females [16]. This could be because, men frequently stay out of their homes late into the night and carry out more outdoor activities like leaving their homes very early in the morning before dawn to go to their farms and often have to spend several days living in their farmhouses, as such, exposing themselves to the mosquito bites. Male children also play outdoors in the early evenings more often than female children and, are, therefore, more exposed to mosquito vectors as found elsewhere [16].

This current study has found that age is associated with being tested positive for malaria. Similar findings have been reported elsewhere [16, 17]. Participants in rural areas are more likely to test positive for malaria as compared to those who live in urban areas. The odds of testing positive for malaria also decreased significantly among the employed and self-employed compared to the unemployed. This conforms to the finding of a study conducted in Cameroon which reported that being unemployed (p = 0.025), living in a rural area (p = 0.013) and the presence of bushes around homes (p = 0.002) were significant risks factors associated with malaria infection [16]. This association among the unemployed is in concordance with the observation that poverty and socioeconomic status of individuals can lead to an increase in malaria prevalence [18] because, the poorest people often have limited access to health services, particularly in rural settings.

### Malaria treatment

Artemisinin-based combination therapy (ACT) was recommended and deployed in 2005 as most efficacious treatment of comfirmed uncomplicated falciparum malaria [19]. According to Ghana's current antimalarial policy, artesunate-amodiaquine (AA) is the first-line drug for uncomplicated malaria with artemether-lumefantrine (AL) and dihydroartemisinin-piperaquine as alternatives for those who cannot tolerate AA [20]. In this current study, malaria treatments given to malaria-positive patients were AL, dihydroartemisinin-piperaquine and artesunate injection was given. Though all the patients were treated with ACT, AA was not used as first-line but AL. This is similar to a study conducted by [21] on direct observation of outpatient management of malaria in Ghana where almost all patients diagnosed with malaria were treated with AL. This finding may be due to stock out of AA, personal choice and fear of adverse effects.

## Conclusion and recommendation

Overall, the study found that the prevalence of malaria was 20.9%. This prevalence is relatively lower as compared to other studies conducted in other settings. A significant proportion of those who tested positive for malaria was not given any form of treatment at all. Factors found to influence malaria infection in the Shai-Osudoku District Hospital include participant’s age, sex, place of residence, religious affiliation, occupation and marital status. The findings of this study showed that malaria remains a serious public health problem in the Shai Osudoku District Hospital, Ghana. The information obtained from this study can guide the implementation of malaria prevention, control and elimination strategies in Ghana.

The study recommend that the Public Health Unit of the hospital and the District Health Directorate should organize community education and sensitization programs within the district on the effects of malaria, especially among those living in rural areas. The District Health Directorate should also engage in the use and distribution of long-lasting insecticidal nets (LLINs) to individuals in the district especially those in rural communities.

## Data Availability

All relevant data supporting the conclusions of this article are included within the article. Any additional information is available from the corresponding author upon reasonable request.

## Abbreviations

AOR: Adjusted odds ratio
ASAQ: Artesunate/Amodiaquine
CHPS: Community-based health planning services
ERC: Ethical Review committee
ITNs: Insecticide Treated Nets
KAP: Knowledge Attitude and Practices
LI: Legislative instrument
LLINs: Long lasting insecticidal nets
MMDAs: Metropolitan Municipal and District Assemblies
NMCP: National Malaria Control Programme
RBM: Roll Back Malaria
SOD: Shai-Osudoku District
SODH: Shai-Osudoku District Hospital
THD: Tombel Health District
UHAS: University of Health and Allied Sciences
WHA: World Health Assembly
WHO: World Health Organization
